# Integration between radiation shielding performance, structural evolution, and mechanical features of co-doped sodium phosphate glasses

**DOI:** 10.1038/s41598-025-33307-w

**Published:** 2026-01-14

**Authors:** Nermin A. Abdelhakim, Rizk Mostafa Shalaby, A. M. Abdelghany, M. Mitwalli, A. H. El-Farrash, Abdelmoneim Saleh

**Affiliations:** 1 Metal Physics Research Laboratory, Physics department, Faculty of Science, Mansoura University, P.O. Box: 35516, Mansoura, Egypt; 2 Spectroscopy Department, Physics Research Institute, National Research Centre, 33 Elbehouth St, Giza, 12311 Egypt; 3 Physics Department, Faculty of Science, Mansoura University, Mansoura, 35516 Egypt; 4 Basic science department, Higher Technological Institute, 10th of Ramadan City 228, Egypt; 5 Faculty of Health Science Technology, Mansoura National University, Gamasa, Egypt

**Keywords:** Radiation shielding, Ionizing radiation, Mechanical characteristics, Niobium-manganese co-doped sodium phosphate glasses, Biophysics, Materials science, Physics

## Abstract

This study investigates the structural, mechanical, and radiation protection behavior of sodium phosphate glasses modified with 30 mol% Nb₂O₅ and varying MnO concentrations (0–7.5 mol%). Seven glass samples (S1–S7) were synthesized via the melt-quenching technique and characterized using FTIR, XRD, Vickers hardness testing, and MCNP simulations combined with XCOM theoretical calculations. FTIR analysis revealed that Nb₂O₅ primarily adopts octahedral coordination (NbO₆), acting as a network modifier, while MnO exhibits dual roles: Mn²⁺/Mn³⁺ ions modify the phosphate network at lower concentrations and participate in structural unit formation at higher concentrations. XRD confirmed the amorphous nature of all glasses. Mechanical testing demonstrated enhanced Vickers hardness (749–1800 MPa) with increasing MnO content, attributed to improved network rigidity. Radiation shielding evaluations highlighted superior gamma-ray attenuation for the S7 sample (7.5 mol% MnO), exhibiting the highest mass attenuation. coefficient (µ/ρ), lowest half-value layer (0.019–9.421 cm), and optimal radiation protection efficiency (100% at 30–100 keV). Neutron attenuation analysis revealed S7’s macroscopic removal cross-section (Σ_r_ = 0.0956 cm⁻¹) outperformed conventional materials like concrete. The glasses demonstrated good stopping power and predicted range for protons and alpha particles with denser compositions (S7) showing enhanced charged particle attenuation. These findings position Nb₂O₅-MnO co-doped sodium phosphate glasses, particularly S7, as promising candidates for radiation shielding in medical, nuclear, and aerospace applications, combining mechanical durability with multi-radiation protection capabilities.

## Introduction

 Ionizing radiation, including X/Gamma-rays, neutrons, and charged ions, is increasingly used in various industries including radiation treatment, medical physics, and nuclear power plants. However, prolonged exposure to these radiations can lead to mutations, cancer, radiation illness, and even death. To effectively utilize these radiations in diverse applications, radiation physicists are seeking superior γ-ray shielding materials. Concretes are often used as shielding materials due to their affordability and accessibility. However, the changing moisture in concrete due to temperature gradients can lead to errors in attenuation coefficient estimations^1–5^.

Scientists are developing a wide range of shielding materials, such as alloys^6–9^, glasses^10–13^, and ferrites^14^. Glasses’ extreme transparency makes them better absorbers of radiations like neutrons and gamma rays than opaque concretes. By adjusting composition and selecting alternative manufacturing techniques, chemical and physical qualities can be improved. The process of making glasses is low-cost and simple to create in large quantities with excellent spatial uniformity.

Sodium phosphate glasses have received a lot of attention in the scientific community because of their unusual features and numerous uses. These glasses belong to the broader category of phosphate glasses, which are formed by the network of phosphorus and oxygen atoms^15–17^. The addition of sodium as a network modifier introduces distinct characteristics that make sodium phosphate glasses particularly interesting for various technological and industrial applications^15,19^. Sodium phosphate glasses are generally characterized by their low melting and softening temperatures, high thermal expansion coefficients, and high ultraviolet and far-infrared transparency^19^.

The fundamental building block of sodium phosphate glass is the phosphate tetrahedron (PO4) at which central phosphorus atom is covalently bonded to four oxygen atoms, forming a tetrahedral configuration connected through bridging oxygen atoms, creating the glass network. The addition of sodium ions (Na^+^) modifies this network by breaking some bridging P-O-P bonds forming non-bridging oxygen atoms^15,16^. Based on the sodium content, these glasses can be classified as Ultraphosphate glasses (0 < x < 0.5), Metaphosphate glasses (x = 0.5), Polyphosphate glasses (0.5 < x < 0.67). Therefore, the varying sodium content significantly influences the properties of the glass including glass transition temperature, melting point, thermal expansion coefficient, chemical durability, ionic conductivity, and viscosity^16,20^.

The unique properties of sodium phosphate glasses make them suitable for a wide range of applications across various fields including optical devices^21^, biomedicine^22^, nuclear waste immobilization^23^, solid-state batteries^24^, sealing materials^25^, laser technology^26^, and radiation shielding^27^.

The incorporation of niobium pentoxide (Nb_2_O_5_) into sodium phosphate glass has been a subject of significant interest in materials science and engineering. Niobium, a transition metal, introduces unique structural modifications and property enhancements when added to the phosphate glass network resulting in a complex interplay of structural changes and property modifications that significantly alter the characteristics of the base glass. Nb_2_O_5_ primarily acts as an intermediate oxide which means that niobium can play dual roles within the glass network and the exact role of niobium depends on its concentration and the overall glass composition^28,29^.

At lower concentrations, niobium tends to enter the glass network as a modifier, occupying interstitial sites and forming ionic bonds with non-bridging oxygen atoms. However, as the concentration increases to higher levels, such as 30%, a significant portion of the niobium ions begin to participate in the network formation. In this role, niobium forms NbO_6_ octahedra that can connect with the existing phosphate tetrahedra, leading to a more complex and interconnected glass structure. Therefore, introduction of niobium may result in network modification, coordination change, bond strength alteration, and phase separation resistance^30,31^.

The addition of Nb_2_O_5_ and MnO to sodium phosphate glass was expected to significantly alters its physical characteristics and enhances its radiation shielding properties. These modifications result in a glass system with unique features that make it suitable for various specialized applications, particularly in environments where radiation protection is crucial. The incorporation of Nb_2_O_5_ and MnO also expected to increases the density of the glass. Additionally, the increase in both Nb_2_O_5_ and MnO tend to enhance the hardness of the glass. The increased network connectivity due to Nb_2_O_5_ results in a more rigid structure, leading to higher Vickers hardness values.

## Experimental techniques

### Glass preparation

Seven glasses in the system *x*MnO-(50-*x*)P_2_O_5_-30Na_2_O-20Nb_2_O_5_, *x* up to 7.5 mol% were successfully prepared via traditional melt quenching route. Manganese oxide and niobium oxide used as received while sodium oxide obtained from their carbonate partner and phosphorus pentoxide obtained from their respective ammonium dihydrogen orthophosphate. All chemicals supplied by Sigma Aldrich company. Sample nomination and composition was listed in Table 1.

The chemicals were mixed thoroughly and placed in a 100 ml porcelain crucible, containing approximately 0.5 mol of the glass batch. The furnace adjusted at about 400 °C for about 2 h until all carbonate and NH_3_ completely evolved, the furnace was then raised gradually to 1200 °C for another 2 h. the melt rotated occasionally at fixed time intervals to obtain homogenous and bubble free samples. The melts were then poured into a heated stainless-steel mold of required dimensions and allowed to cool slowly to the room temperature.

The experimental glass density at room temperature (*D*_*exp*_) was determined by using the Archimedes principle by weighing the sample both in air (*wt*_*air*_) and after immersion in xylene (*wt*_*xylene*_), of density (*D*_*xylene*_=0.863 g/cm^3^). Table 1)

**Table 1:** Sample nomination, composition and density.

Sample	Na_2_O	Nb_2_O_5_	*P*_2_O_5_	MnO	Density (g/cm^3^)
S1	30	20	50	0	2.7960
S2	30	20	49	1	2.8258
S3	30	20	48	2	2.8556
S4	30	20	47	3	2.8854
S5	30	20	46	4	2.9152
S6	30	20	45	5	2.945
S7	30	20	42.5	7.5	3.0195

### FTIR spectrometer measurements

The structural composition of the glass was analyzed using a Bruker Vertex 70 FTIR spectrometer. The measurements recorded within the wavenumbers extended from 4000 to 400 cm^− 1^. To prepare the sample for analysis, pulverized fine powder mixed with KBr (1:100) compressed under 5 tons/cm^2^ of pressure to create a transparent disk suitable for testing.

### XRD characterization

In addition, a Bruker TM-D8 diffractometer (XRD) examination employing materials Powder diffractometer of the Advanced Series that employs Cu Kα radiation (λ = 1.5406 Å) was used for measurements at room temperature. It was operated at 40 kV and 30 mA, with a scan speed of 2° per minute in the 2θ range of 10 ~ to 80.

### Hardness measurements

**Fig. 1:**
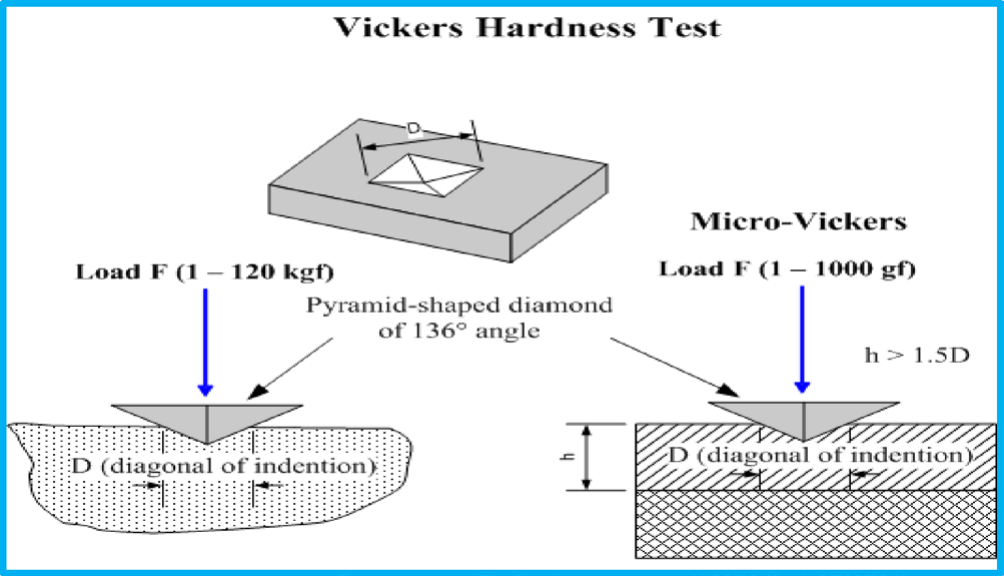
Schematic diagram of Vickers pyramid diamond indenter.

One of the common techniques for determining the hardness of materials, particularly those with extremely hard surfaces, is the digital Vickers microhardness tester model (FM-7). Micro-indentation creep technology’s primary benefits are ease of use, speed, non-destructive nature, and accuracy. Furthermore, a single kind of indenter may be utilized for various kinds of surface treatments^32,33^. Despite the fact that they are more precise and adaptable in determining the hardest and softest materials under varying pressures. A a pyramid-like diamond, as shown in schematic Fig. 1, applies standard pressure to the surface for a specific period of time. The indenter is a square pyramid with a 136-degree angle formed at its peak, where the opposing sides meet. Using a load of 10 gf, diamond pressure is applied to the material’s surface, and the impression size is examined under a microscope. The following formula is used to determine the number of vickers (H_V_):

1 $$Hv = \frac{{2F~\mathrm{Sin} ~\frac{{136^{o} }}{2}}}{{d^{2} }}$$

So that Hv=$\:1.854\:\frac{\mathrm{F}}{{\mathrm{d}}^{2\:}}\:\mathrm{n}\mathrm{e}\mathrm{a}\mathrm{r}\mathrm{l}\mathrm{y}\:\:\:$.

With (F) is the load applied (Kgf), d is the mean of two diagonals d1 and d2 (mm).

### Radiation shielding calculations

#### MCNP5 code

**Fig. 2:**
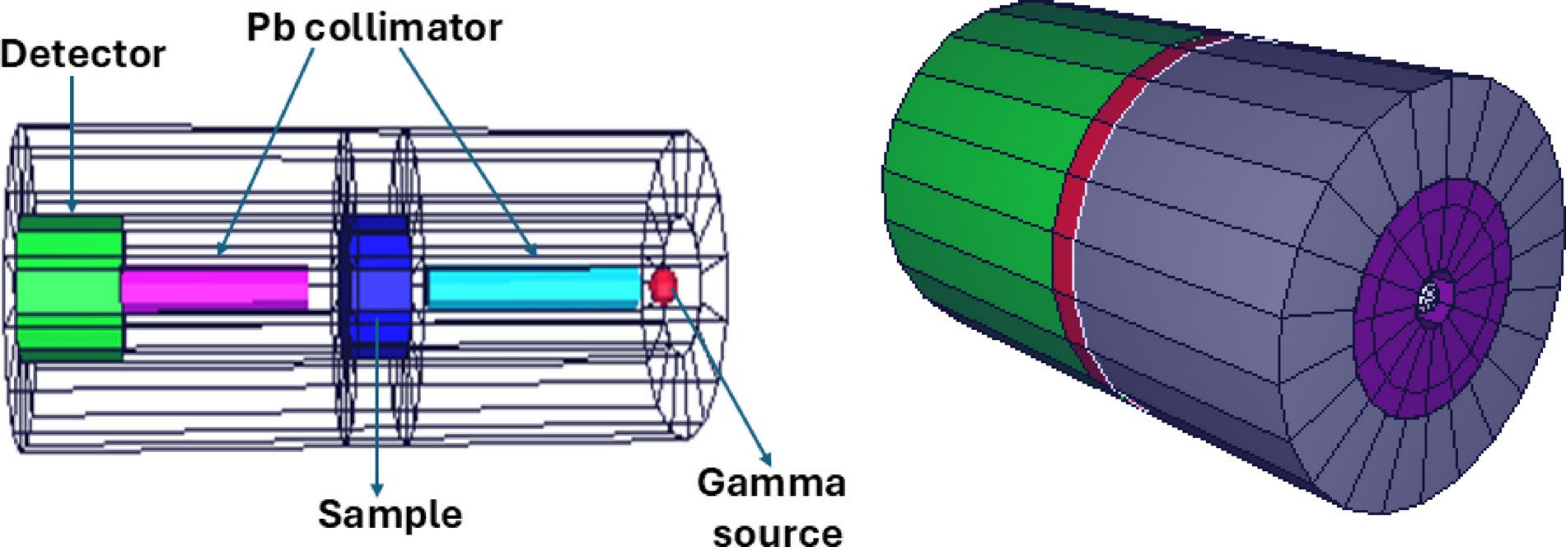
The MCNP5 Visualization Editor’s modeling setup for determining the gamma-ray attenuation factors.

Monte Carlo simulation is a powerful mathematical method used to solve complex physical issues. It mimics experimental settings by considering cross-section values, databases, and equipment characteristics. This technique is particularly useful for determining the shielding effectiveness of materials against radiation. In this study, the authors determined the mass-attenuation coefficient (µ_m_) for prepared glasses using the Monte Carlo technique. The µ_m_ values were obtained using the MCNP5 code, which is based on the Lambert-Beer equation. The 3D configuration used in the MCNP5 simulation includes a lead collimator, a point isotropic source, the sample, an F4 tally mesh for detection, and lead blocks to reduce dispersed radiation (Fig. 2). The F4 tally measured the photon flux (MeV cm²s⁻¹), with the sample situated between the mesh and the source. The cell architecture of the MCNP5 code varies depending on material characteristics, which are defined by the fractions of components. The simulations used a high particle count (10^8^) to assure accurate results^34,35^.

For calculating the differences within the µ/ρ values based on the XCOM tool and the MCNP5 simulating code, the percentage of relative variation (RD%) can be determined with the formula that follows^36^:

2 $$\:RD=\left|\frac{(\mu\:/\rho\:)MCNPX-(\mu\:/\rho\:)XCOM}{(\mu\:/\rho\:)MCNPX}\right|\times\:100$$

Furthermore, the mass attenuation coefficient (µ_m_), which assesses an absorber’s ability to attenuate photons of a specific energy, was calculated using the WINXCOM program. This made it possible for scientists to investigate the contemporary glass samples’ gamma-ray shielding capabilities. The range of photon energies covered by the mixing rule was 0.015 to 15 MeV^37^.

3 $$\:{{\upmu\:}}_{m}={\sum\:}_{i}{w}_{i}{\left(\frac{{\upmu\:}}{\rho\:}\right)}_{i}$$

where (µ/ρ)_i_ were calculated with the WINXCOM program of the i^th^ composition element. The half value layer (HVL) is a crucial element in assessing gamma-ray shielding characteristics and measuring the degree of gamma-radiation penetration into the attenuator sample. HVL is the thickness at which the incident photon intensity is divided in half. It is a measure of the energy-dependent parameter and penetrating capabilities of photons. HVL values were computed using the linear attenuation coefficient of the sample µ (cm^− 1^) according to the following formula^37^:

4 $$\:HVL=\frac{\mathrm{l}\mathrm{n}\left(2\right)}{\mu\:}$$

Several absorption characteristics, including effective atomic number (Z_eff_) and electron density (N_el_), are also determined using the µ_m_ values and may be computed using the following formulas^36^.

5 $$\:{Z}_{eff}=\frac{{{\upsigma\:}}_{t,a}}{{{\upsigma\:}}_{t,el}}=\frac{\sum\:_{i}{n}_{i}{A}_{i}{(\mu\:/\rho\:)}_{i}}{\sum\:_{i}{n}_{i}{A}_{i}/{Z}_{i}{(\mu\:/\rho\:)}_{i}}$$

The following formula may be used to get the material’s electron density, sometimes referred to as the “number of electrons per gram,” or N_el_, using µ_m_ and σ_t_^37^.

6 $$\:{N}_{el}=\frac{{\mu\:}_{m}}{{{\upsigma\:}}_{e}}=\frac{{Z}_{eff}{N}_{A}\sum\:_{i}{n}_{i}}{M}$$

Z_i_ is the atomic number of the element, and M is the sample molar mass. The following formula, which accounts for the initial and attenuated photon intensity (I_0_ and I), may also be used to calculate the radiation protection efficiency (RPE) of the resultant glass samples^38^:

7 $$\:RP=\left(1-\frac{I}{{I}_{0}}\right)\times\:100$$

### Macroscopic fast neutron effective removal cross sections (∑_R_)

The study evaluates the potential of using current glass samples as fast neutron attenuators by evaluating their neutron shielding capacities using the “effective macroscopic removal cross section” (∑R in cm^− 1^). The effective elimination cross section for neutron energy is consistent between 2 and 12 MeV, with the majority of neutrons produced during uranium fission having energies between one and two MeV. Low-Z element density and composition play a significant role in radiation protection against neutrons and gamma rays. The effective fast neutron removal cross section (∑R (cm-1)) of a glass sample can be calculated by considering the partial densities and cross sections of the component elements^39,40^.

8 $$\:{\:\sum\:}_{R}={\sum\:}_{wi}{\left({\sum\:}_{R/\rho\:}\right)}_{i}$$

where ∑_(R/ρ)_ (cm^2^/g) represent and the cross-section for mass removal of the i^th^ component.

### Parameters of shielding for heavy charged ions (alpha particles and protons)

The mass stopping power (MSP) of a charged particle is the average energy lost when it passes through a substance, such as a proton or alpha particle. This is determined by the energy lost in collisions per mass of dense material. Nuclear physics techniques and understanding of particle-matter interactions depend on identifying heavy charged particles that transmit and absorb at varying energies. The projected range, or the thickness of the medium required to stop the particle, measures the attenuation of ions by a given medium. This affects the ability of heavy charged particles to pass through absorbent materials and is closely related to energy and material properties. SRIM software was used to calculate MSP and PR values for protons and alpha particles^41^.

## Results and discussion

### X-ray diffraction analysis

**Fig. 3:**
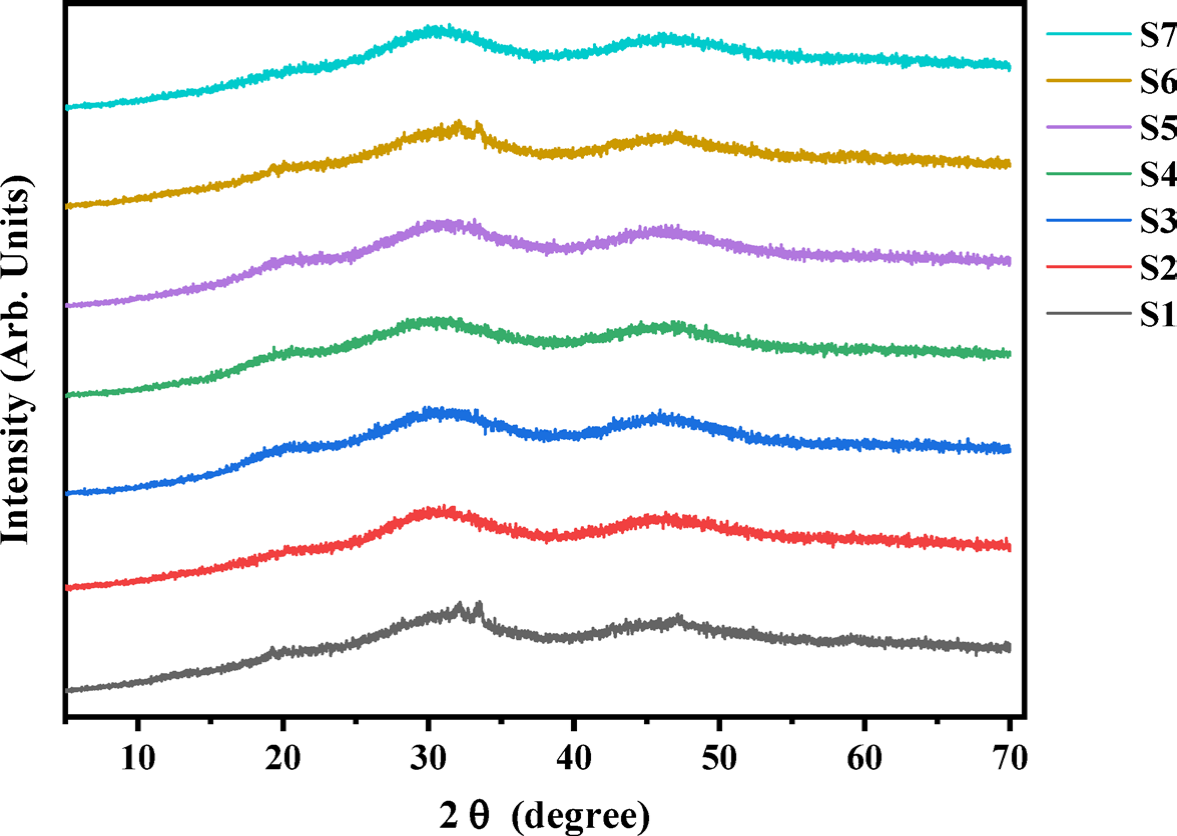
The XRD patterns of glass sample for varying Nb contents.

Figure 3 reveals the XRD patterns of Nb_2_O_5_ doped-phosphate glasses. These XRD patterns demonstrate two broad humps without any sharp peaks, signifying that the amorphous nature of these glasses. The first hump observed in these glasses at 2θ = 30^o^, which is related to the amorphous character of these glassy samples, while the second hump observed at 2θ = 46^o^ is most likely due to the niobium ion glass network in its amorphous form in this range of compositions.

###  FTIR spectral data

The addition of 30% Nb_2_O_5_ to sodium phosphate glass represents a significant modification that transforms the base glass into a new material with enhanced properties. This niobium-modified sodium phosphate glass finds potential applications in various fields, including optical devices, radiation shielding materials, chemical-resistant coatings, and specialized glass-ceramics. The unique combination of improved durability, altered optical properties, and enhanced thermal stability opens new possibilities for tailoring these glasses for specific technological requirements.

**Fig. 4:**
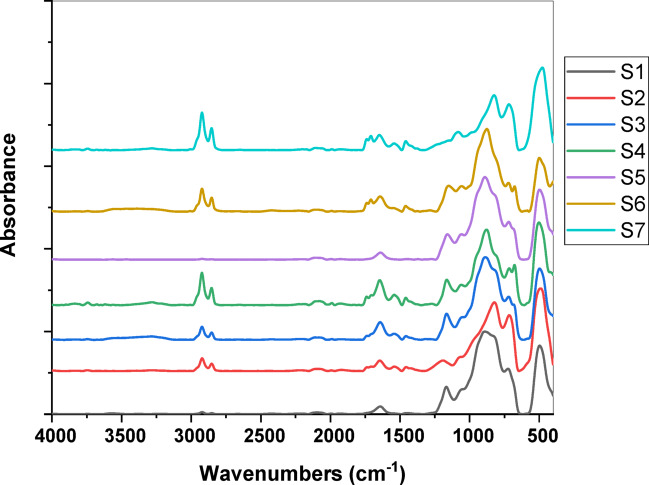
FTIR absorption spectral data of Nb_2_O_5_ doped- phosphate glasses.

Figure 4 reveals FTIR absorption spectral data of the studied glasses that reveal a complex structural arrangement within the glass system as MnO content varies. At lower MnO concentrations, the spectrum shows characteristic features of phosphate networks modified by niobium and sodium. The band at 400 cm^− 1^ (shoulder) can be attributed to the bending modes of O-P-O bonds, while the intense peak at 510 cm^− 1^ corresponds to O-P-O deformation vibrations. The prominent peak at 675 cm^− 1^, along with the shoulder at 715 cm^− 1^, indicates the presence of Nb-O bonds in NbO_6_ octahedra, suggesting that niobium predominantly acts as a network modifier in octahedral coordination^15,16,42^.

The broad intense peak centered at 900 cm^− 1^ is characteristic of the asymmetric stretching vibrations of P-O-P linkages in the phosphate network, while the shoulders at 950 and 1065 cm^− 1^ can be assigned to the symmetric and asymmetric stretching modes of PO_4_ tetrahedra, respectively. The small peak at 1170 cm^− 1^ likely corresponds to P = O terminal bonds. The presence of weak bands at 2850 cm^− 1^ and 2925 cm^− 1^ might be attributed to hydroxyl groups or atmospheric moisture absorbed by the samples^15–17,42^.

As the MnO concentration increases to 7.5%, significant structural modifications occur as shown in Fig. 4. The splitting of the broad band in the 700–900 cm^− 1^ region into two distinct peaks at 720 and 815 cm^− 1^ suggests a transformation in the phosphate network. This change indicates that manganese ions are actively participating in the network modification, likely entering the glass structure in both Mn^2+^ and Mn^3+^ states. The appearance of multiple small peaks at higher wavenumbers (1460, 1540, 1650, 1710, and 1740 cm^− 1^) indicates the formation of more complex structural units, possibly due to the interaction between manganese and phosphate groups [].

Nb_2_O_5_ primarily acts as an intermediate oxide in the glass network. Niobium typically adopts octahedral coordination (NbO_6_), which can either strengthen the glass network by forming Nb-O-P bonds or act as a network modifier by creating non-bridging oxygens^31,45^. The role of MnO is more complex and concentration-dependent. At lower concentrations, Mn^2+^ ions likely act as network modifiers, occupying interstitial sites. However, at higher concentrations (7.5%), the structural changes evident in the FTIR spectra suggest that manganese ions may partially enter the glass network, possibly in both Mn^2+^ and Mn^3+^ oxidation states, leading to the formation of MnO_4_ and MnO_6_ structural units. The observed evolution in the FTIR spectra confirms that the formation of NbO₆ octahedra and the mixed oxidation states of Mn ions (Mn²⁺/Mn³⁺) play a pivotal role in modifying the glass network. The NbO₆ units act as cross-linking centers, introducing additional Nb–O–P bonds that strengthen the phosphate framework and restrict structural mobility. Concurrently, the presence of Mn²⁺ ions at lower concentrations contributes to network depolymerization and improved densification, while Mn³⁺ ions at higher concentrations promote the formation of MnO₆ structural units that enhance connectivity and rigidity. This progressive structural reinforcement explains the observed increase in glass density and hardness, as well as the improved attenuation of ionizing radiation due to the higher effective atomic number and electron density associated with Nb and Mn incorporation. This dual role of manganese contributes to the observed changes in glass network structure and the appearance of new vibrational modes at higher concentrations.

###  Hardness indentation and micro-creep dependence

**Fig. 5:**
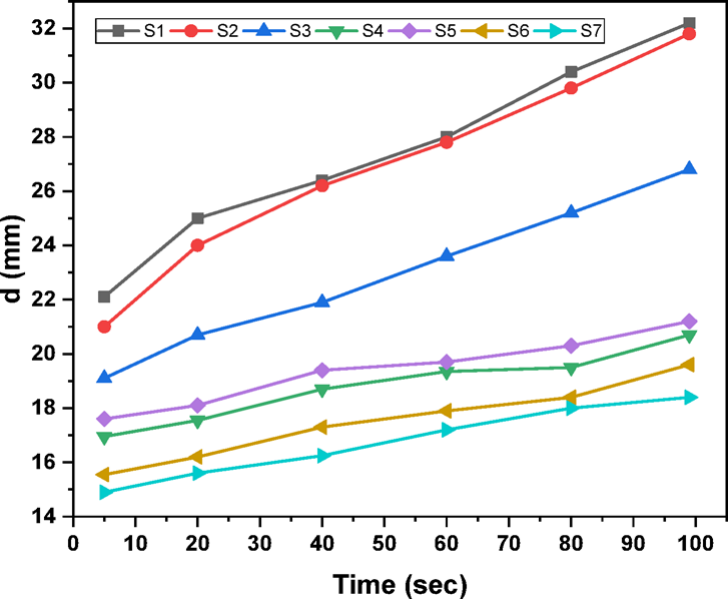
Variation of indentation length with time at constant load 25 gf.

**Fig. 6:**
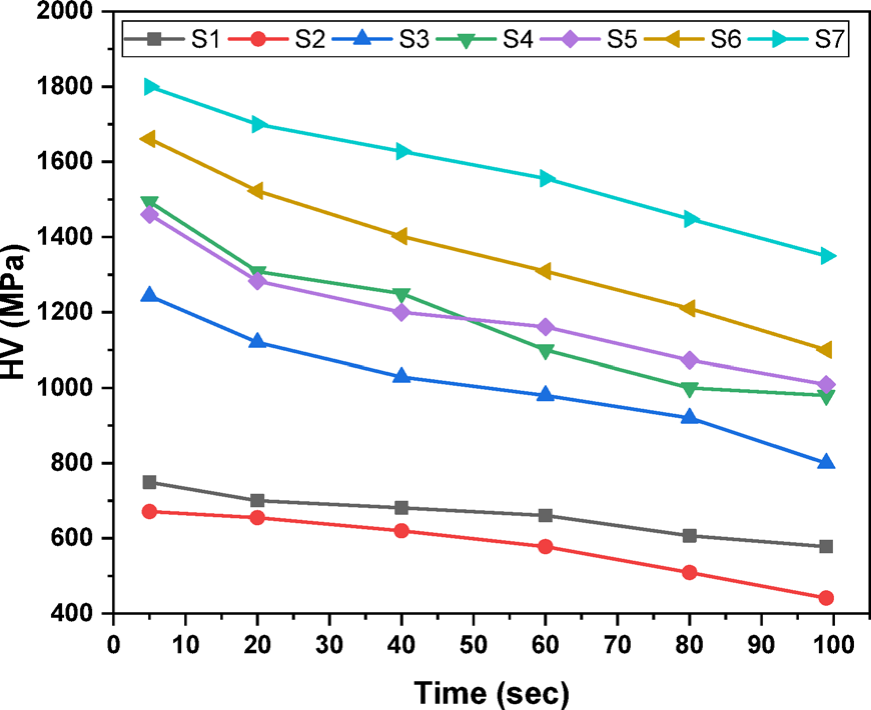
Variation of hardness with time at constant load 25 gf.

**Fig. 7:**
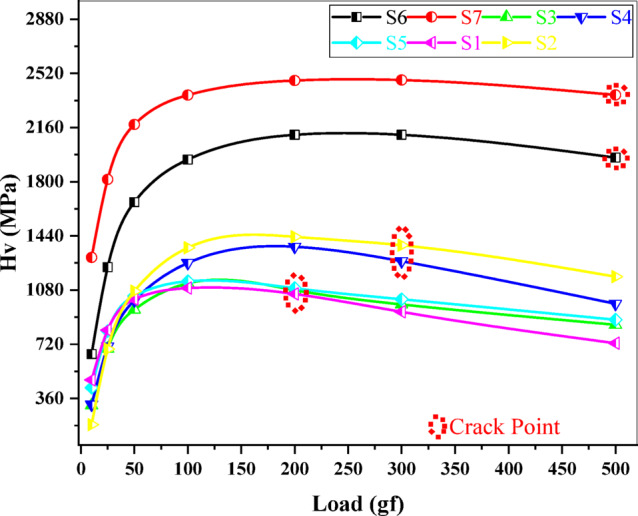
Variation of hardness with load at constant time 5 s.

An indentation test of hardness determines the resistance to plastic deformation by pushing a very little rigid indenter into the surface with a predetermined load and measuring the result. Figure 5 depicts the relationship between length and penetration time under a constant load of 25 gf. This graph demonstrates that as the loading time increases, so does the penetration length. Figure 6 shows the Vickers hardness variation as a function of indentation time^46^. According to Fig. 6, the hardness decreases as the dewell duration increases. The hardness number HV is defined as the load on the indenter divided by the contact area between the indenter and the material, which is commonly expressed in kg/mm^2^. The plastic restraint coefficient remains constant even when the indentation is not a plane strain, hence a reasonable estimate is HV ≈ 3Y, where Y is the yield strength^47^. Table 2 displays the average hardness and yield strength values. These results show that increasing the MnO content greatly increased the hardness and yield strength. Record the relationship between hardness and load at constant time 5 s, as illustrated in Fig. 7. Based on this graph, we can observe that the hardness increases with increasing load until it hits the crack point, at which time the hardness turns down. The crack point occurred at load 500 gf for S6 and S7 samples which indicated that these samples are more resistant to cracking that other samples. Equation (11) was used to predict the power law creep behavior of the samples at room temperature and to get the stress exponent (n)^48^.

11 $$\:\mathrm{n}={\left[\frac{\partial\:\mathrm{l}\mathrm{n}\dot{\mathrm{d}}}{\partial\:\mathrm{l}\mathrm{n}{\mathrm{H}}_{\mathrm{v}}}\right]}_{\mathrm{d}}$$

**Fig. 8:**
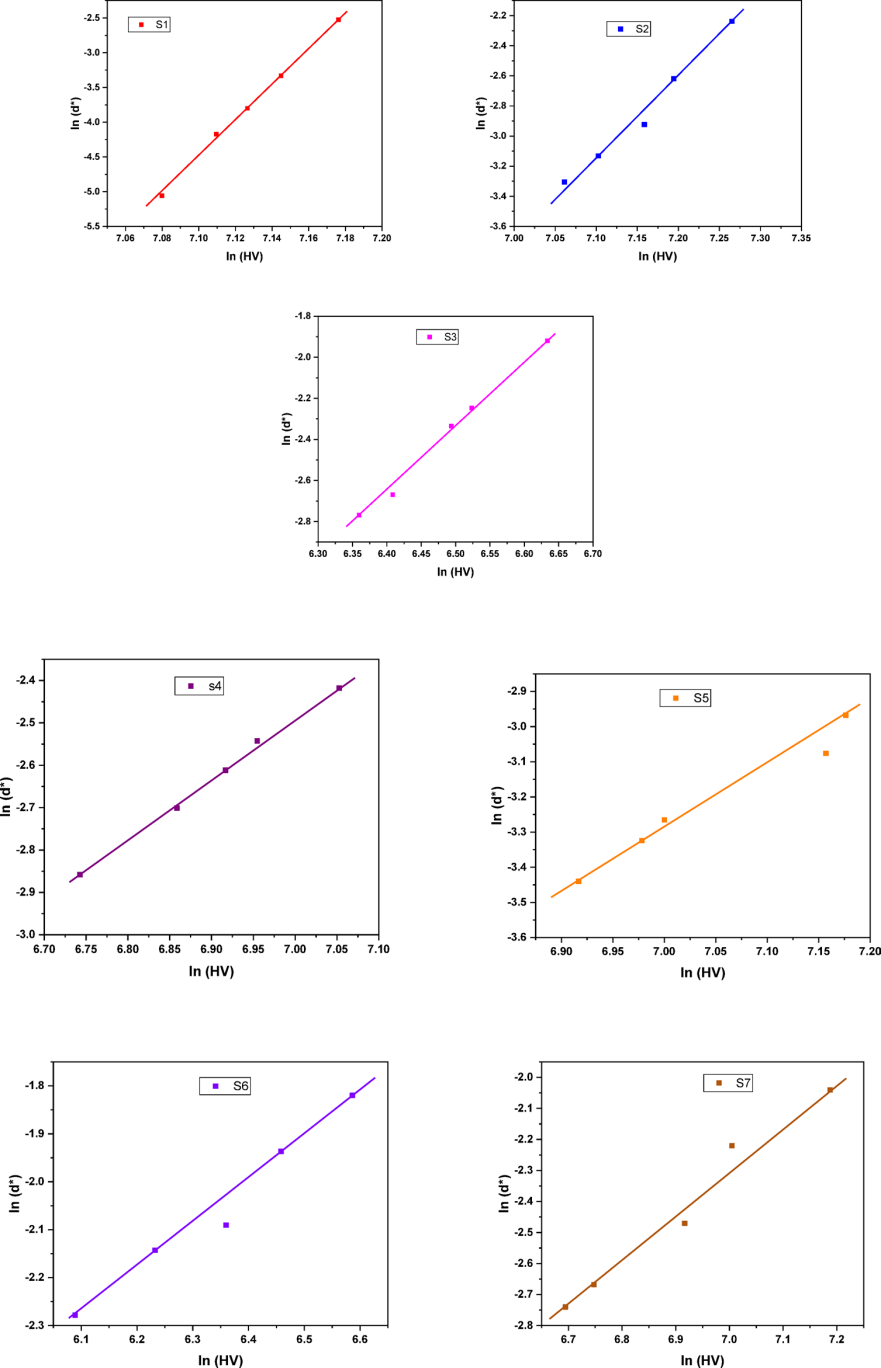
ln-ln of the Vickers hardness numbers against the dwell time of indentation at load 25 gf.

A log-log scale plot of variation rate of indentation length (d*) for each sample and its Vickers hardness number are presented in Fig. 8. To determine the mechanisms governing the deformation process, use the stress exponent (n) shown in Table 2. The values of *n* ≈ 1 are linked to diffusion creep, *n* ≈ 2 to grain boundary sliding, and *n* ≈ 5–7 to dislocation movement^49^. Microstructural characteristics are the cause of changes in stress exponent values. Table 2 verifies our findings by showing that increasing MnO content increases yield strength values. The significant improvement in hardness and yield strength with increasing MnO content can be directly attributed to the structural evolution identified through FTIR analysis. The integration of NbO₆ octahedra and Mn-induced cross-linking leads to a denser, more rigid phosphate network that resists plastic deformation. The synergistic effect of Nb and Mn therefore enhances both elastic recovery and load-bearing capacity. Moreover, the increased network connectivity reduces atomic mobility, which contributes to the lower creep rate observed at higher Mn levels. This structural consolidation not only strengthens the mechanical framework but also supports superior gamma and neutron attenuation, as the denser glass matrix provides more effective interaction sites for radiation absorption.

**Fig. 9:**
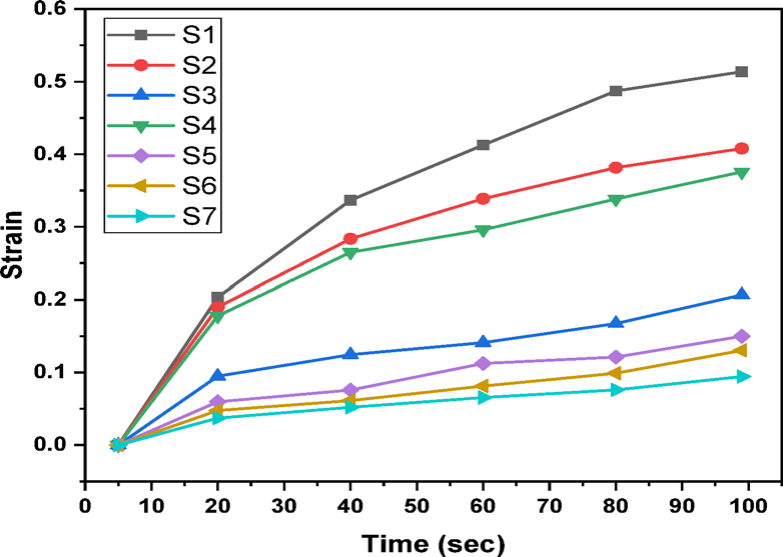
The Indentation creep behaviour.

Plotting strain against indentation time yields a typical indentation creep curve, as Fig. 9 illustrates. The strain increases quickly in the initial part of the curve, from zero to ten seconds after indentation. In the second region, all samples exhibit a steadily expanding region with increasing load. The hardness test, unlike the standard creep test, does not include sample failure because it is essentially a compression test. As a result, the third segment of the curve cannot be recorded. The creep strength was improved by adding more MnO.

**Table 2:** Hardness and stress exponent value at time 5 s.

Sample	HV (MPa)	*n*	Y (MPa)
S1	749.1	5.1	249.7
S2	671.4	5.3	223.8
S3	1243.2	3.1	414.4
S4	1494.9	1.43	498.3
S5	1460.8	1.11	486.9
S6	1661.5	1.46	553.8
S7	1800.4	1.48	600.1

### Radiation shielding features

The MCNP5 simulation code and XCOM computer software were used to estimate the µ/ρ values for the prepared samples at radiation energies ranged from 0.01 to 15 MeV. These results and their relative deviance (RD%) are tabulated in Table 3.

**Table 3:** Mass attenuation coefficients (µ_m_) of the current samples calculated by (ⅰ) XCOM (ⅱ) MCNP5.

Energy (MeV)	S1			S2				S3				S4			
	XCOM	MCNPX	RD	XCOM	MCNPX		RD	XCOM	MCNPX		RD	XCOM	MCNPX		RD
0.015	8.2541	8.6217	4.2636	8.5830	8.7161		1.5266	8.9122	9.2483		3.6345	9.2411	9.432		2.0235
0.020	12.767	13.2432	3.595	12.9150	13.5049		4.3679	13.063	13.3168		1.9056	13.2110	13.457		1.8329
0.030	4.4156	4.4444	0.647	4.4622	4.5161		1.1946	4.5088	4.5463		0.8247	4.5554	4.7555		4.2078
0.040	2.0847	2.1412	2.638	2.1048	2.1639		2.7292	2.1249	2.1352		0.4824	2.1451	2.2151		3.1602
0.050	1.1881	1.2303	3.432	1.1985	1.2171		1.5258	1.2089	1.2164		0.6201	1.2193	1.2557		2.9013
0.060	0.7704	0.7982	3.484	0.7765	0.7947		2.2955	0.7825	0.7911		1.0830	0.7885	0.7932		0.5862
0.080	0.4200	0.4205	0.120	0.4226	0.4364		3.1732	0.4251	0.4389		3.1393	0.4276	0.4439		3.6753
0.100	0.2860	0.2949	3.015	0.2873	0.2999		4.2251	0.2885	0.2937		1.7722	0.2898	0.2924		0.8944
0.200	0.1386	0.1450	4.380	0.1387	0.1397		0.7015	0.1388	0.1417		2.0505	0.1390	0.1433		3.0408
0.300	0.1098	0.1137	3.436	0.1098	0.1127		2.6071	0.1098	0.1152		4.7547	0.1098	0.1127		2.6181
0.400	0.0955	0.0981	2.584	0.0955	0.0962		0.6824	0.0955	0.0988		3.3284	0.0955	0.0993		3.8504
0.500	0.0862	0.0890	3.150	0.0861	0.0870		0.9933	0.0861	0.0871		1.1251	0.0861	0.0896		3.9212
0.600	0.0792	0.0794	0.245	0.0792	0.0819		3.3680	0.0791	0.0830		4.6628	0.0791	0.0811		2.4225
0.800	0.0691	0.0696	0.703	0.0691	0.0722		4.3406	0.0691	0.0692		0.2085	0.0690	0.0722		4.4322
1.000	0.0620	0.0639	3.023	0.0620	0.0636		2.5787	0.0619	0.0643		3.6528	0.0619	0.0634		2.4346
2.000	0.0436	0.0437	0.248	0.0436	0.0440		1.0457	0.0436	0.0437		0.3872	0.0435	0.0457		4.6334
3.000	0.0358	0.0363	1.381	0.0358	0.0358		0.0375	0.0358	0.0362		0.9534	0.0358	0.0367		2.3065
4.000	0.0316	0.0329	4.037	0.0316	0.0324		2.3912	0.0316	0.0321		1.6639	0.0316	0.0331		4.6593
5.000	0.0289	0.0290	0.078	0.0290	0.0302		4.2195	0.0290	0.0298		2.8713	0.0290	0.0291		0.2352
6.000	0.0272	0.0275	1.005	0.0272	0.0283		3.9019	0.0272	0.0278		1.8962	0.0273	0.0274		0.5923
8.000	0.0251	0.0256	1.772	0.0252	0.0255		1.2159	0.0252	0.0254		0.7333	0.0252	0.0263		3.9828
10.000	0.0240	0.0245	1.826	0.0241	0.0249		3.3121	0.0241	0.0243		0.6908	0.0242	0.0247		2.1524
15.000	0.0230	0.0237	3.112	0.0231	0.0236		2.3708	0.0231	0.0235		1.5679	0.0232	0.0240		3.2081

Energy (MeV)	S5			S6				S7			
	XCOM	MCNPX	RD	XCOM		MCNPX	RD	XCOM		MCNPX	RD
0.015	9.5703	10.00032	4.300	9.8992		10.2091	3.036	10.722		10.8578	4.290
0.02	13.36	13.9451	4.196	13.508		13.6492	1.035	13.878		14.0538	1.832
0.03	4.6021	4.808403	4.290	4.6486		4.70214	1.139	4.7652		4.82558	0.924
0.04	2.1652	2.205611	1.832	2.1853		2.26123	3.358	2.2357		2.26403	1.728
0.05	1.2297	1.241173	0.924	1.2401		1.25066	0.844	1.2661		1.28214	4.120
0.06	0.7945	0.808549	1.728	0.80061		0.83501	4.120	0.8157		0.82604	1.101
0.08	0.4301	0.448847	4.168	0.43267		0.43748	1.101	0.4389		0.44455	2.950
0.1	0.2910	0.297742	2.238	0.29235		0.30123	2.950	0.2955		0.29926	2.823
0.2	0.1390	0.144794	3.953	0.13918		0.14322	2.823	0.1394		0.14123	2.206
0.3	0.1097	0.110737	0.873	0.10977		0.11224	2.206	0.1097		0.11117	1.585
0.4	0.0954	0.097713	2.335	0.095406		0.09694	1.585	0.0953		0.09655	1.539
0.5	0.0860	0.09013	4.537	0.086007		0.08735	1.539	0.0859		0.08701	3.708
0.6	0.0790	0.079339	0.360	0.079019		0.08124	2.743	0.0789		0.07993	2.834
0.8	0.0690	0.071661	3.708	0.068971		0.06965	0.985	0.0688		0.06975	3.421
1	0.0618	0.063669	2.834	0.061833		0.06380	3.094	0.0617		0.06253	1.118
2	0.0435	0.045064	3.421	0.043504		0.04394	1.000	0.0434		0.04400	0.296
3	0.0358	0.036222	1.118	0.035812		0.03628	1.294	0.0357		0.03625	3.814
4	0.0315	0.03169	0.296	0.031601		0.03213	1.653	0.0316		0.03201	2.876
5	0.0289	0.030148	3.814	0.029013		0.03032	4.324	0.0290		0.02941	0.505
6	0.0272	0.028089	2.876	0.027304		0.02760	1.091	0.0273		0.02770	3.011
8	0.0252	0.025393	0.505	0.0253		0.02587	2.229	0.0253		0.02570	3.708
10	0.0242	0.024969	3.011	0.024262		0.0246	1.375	0.0243		0.02468	2.834
15	0.0232	0.023305	0.227	0.023314		0.02396	2.726	0.0234		0.02376	3.421

**Fig. 10:**
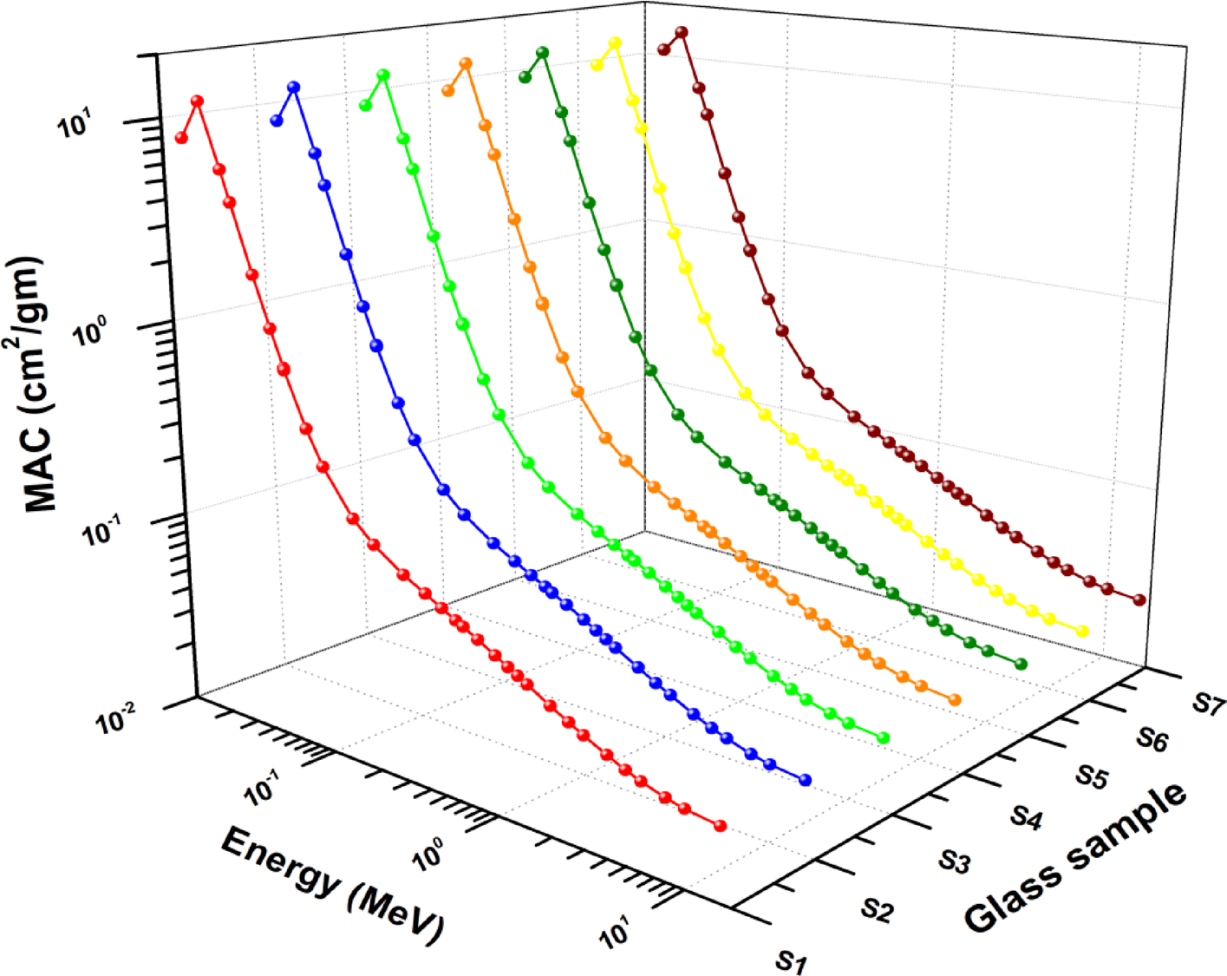
The estimated values of µ_m_ for the prepared glasses using XCOM program and the MCNP5 simulation code.

To completely comprehend how photon energy influences the µ/ρ quantities for the materials used in this experiment, the obtained results are displayed in Fig. 10. The MCNP5 simulation code shows a high correlation between the XCOM and MAC values of the current samples. µ_m_ values decrease with increasing photon energy, and they are correlated with an rise in Mn content. Partial photon interactions that rely on photon energy include Compton scattering (CS), pair production (PP), and the photoelectric effect (PE). The main cause of the sharp decline in µ_m_ values in the low energy range of 0.105–0.15 MeV is the photoelectric effect (PE). µ_m_ values are slightly reduced in the intermediate energy region (0.051–5.0 MeV) due to the CS process. µ_m_ values gradually rise in the high energy area (1.023–15 MeV) due to the PP process. The S7 sample is considered the most suitable for radiation-shielding relative to the glass samples studied.

**Fig. 11:**
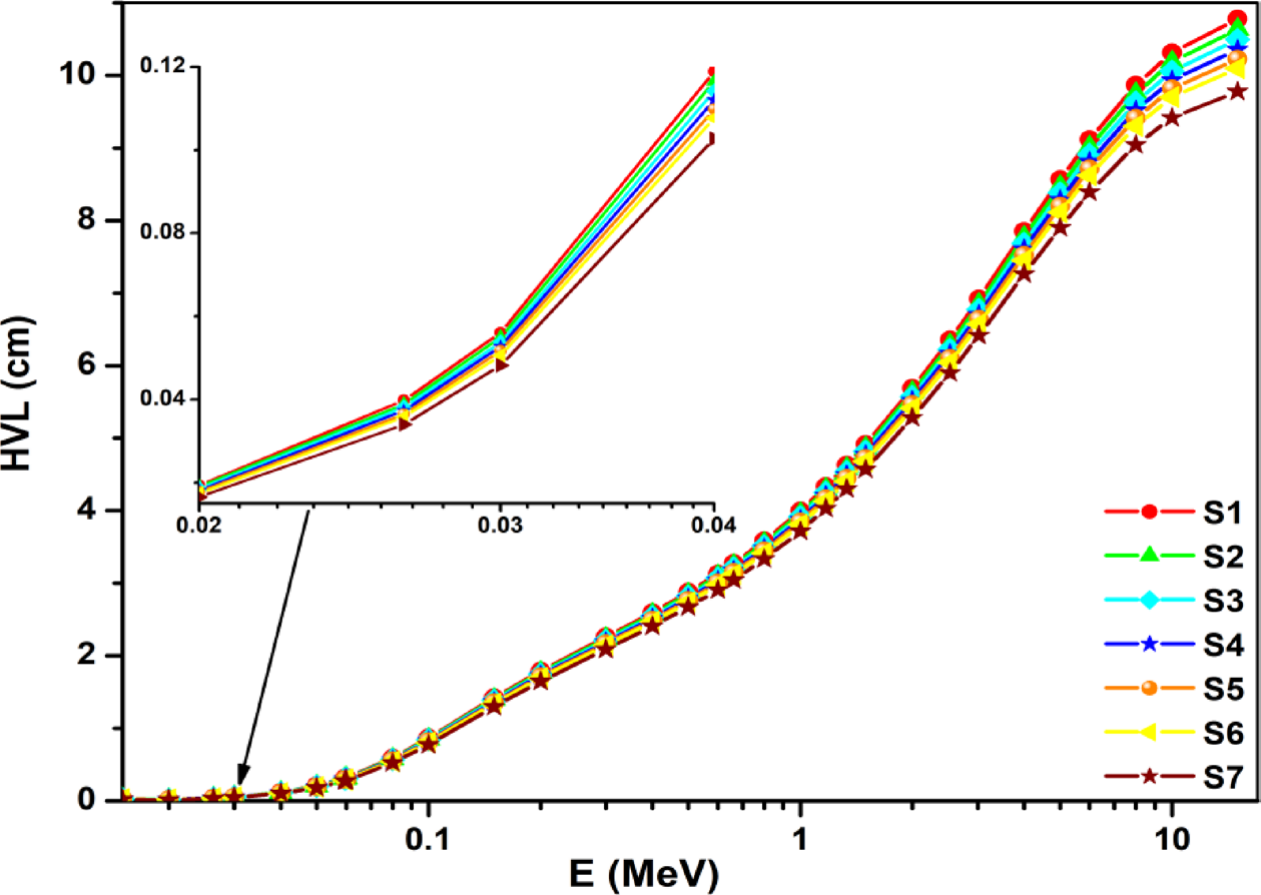
The estimated HVL values of the prepared glasses relative to photon energy.

The HVL results for the prepared glasses are determined using the calculated values of µ_m_ and are shown relative to radiation energy in Fig. 11. For energies below 50 keV, the HVL values are nearly constant, as seen in Fig. 11. The behavior of HVL can be explained in a similar way to how µ_m_ relative to radiation energy was previously explained. The S7 sample had the lowest HVL values, between 0.019 and 9.421 cm, according to the investigation’s findings.

**Fig. 12:**
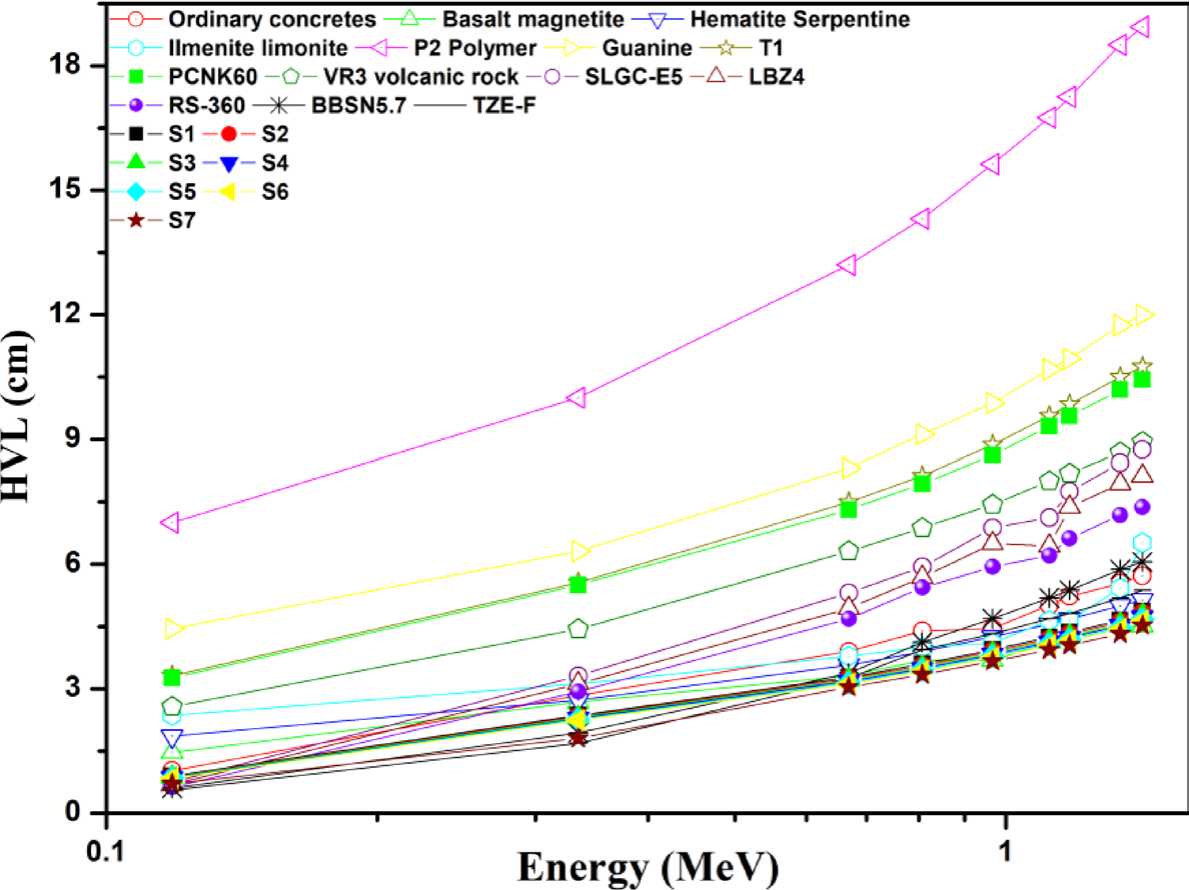
The HVL values of the present glass samples in comparison with those of other frequently used radiation protection materials.

Furthermore, an analysis was conducted to compare the HVL results obtained for the current glass with some common shielding materials and a number of recently researched materials^14^. Figure 12 demonstrates that the S7 galss’s half-value layer (HVL) is substantially lower than the HVL values of all the other prepared samples and the HVL values discovered in previous studies of widely used radiation shielding materials, confirming the suitability of using the current glass as radiation shielding materials.

**Fig. 13:**
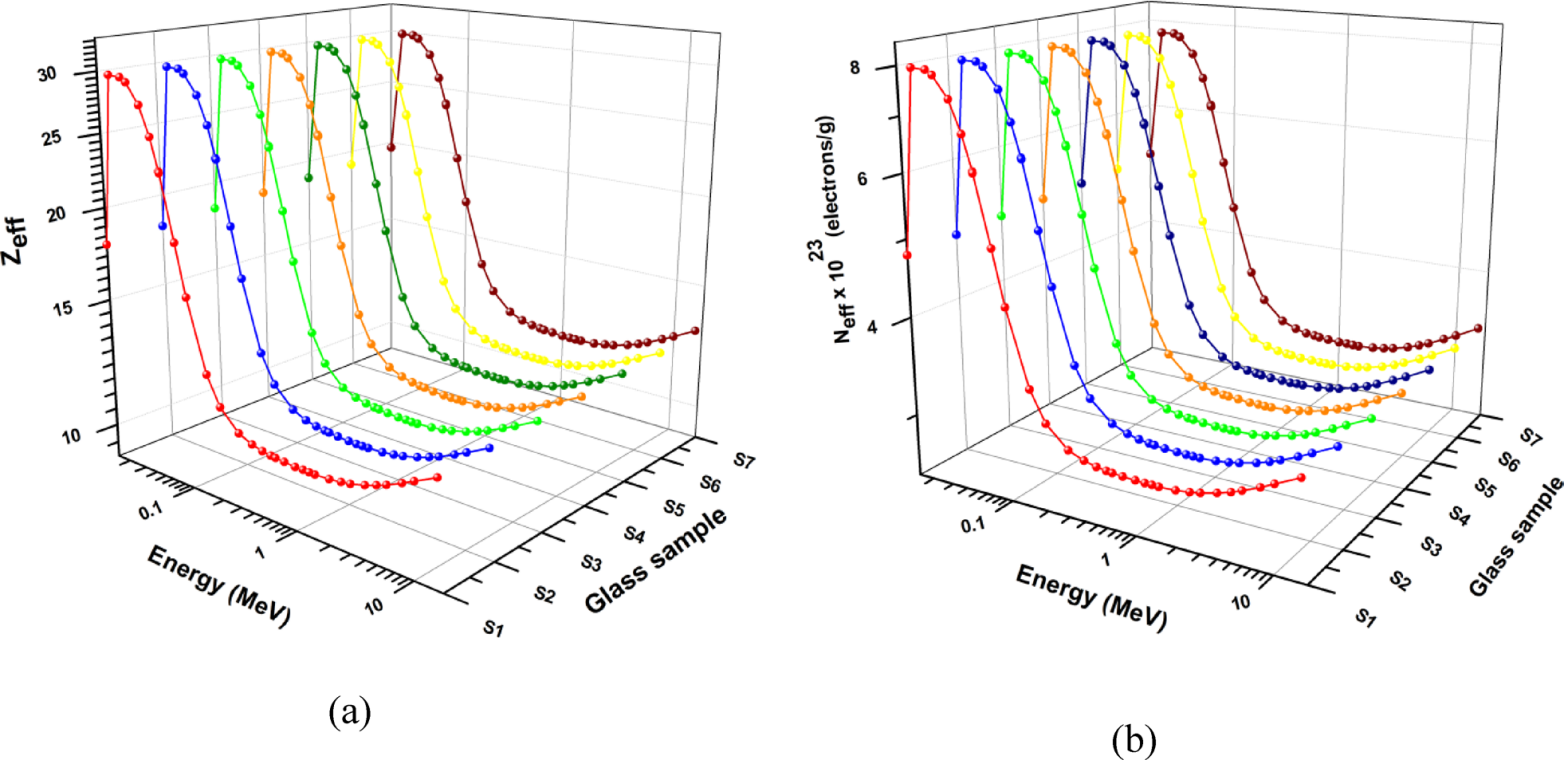
The produced glass’s (**a**) effective atomic number (Zeff) and (**b**) electron density (Nel) relative to photon energy.

The data presented in Fig. 13a, b demonstrate that, for all prepared glasses, the electron density (N_el_) and effective atomic number (Z_eff_) values fluctuate in a similar pattern across the different radiation energy levels.

The data presented in Fig. 13a and b demonstrate that, for all prepared glass, the Z_eff_ and N_el_ values fluctuate in a similar pattern across the different radiation energy levels. A peak is observed at 21 keV because of absorption edge of Mn. Beyond this peak, there is a noteworthy decline in Z_eff_ and N_el_ values as the photon energy increases up to 0.1 MeV, indicating the dominance of the photoelectric effect in this energy range. From 0.1 to 1.5 MeV, the Z_eff_ and N_el_ values exhibit a minor decrease or variation, with the lowest values observed at 1.5 MeV, due to the dominance of the Compton. After then, there is a sharp decline in photon energy until it reaches 1 MeV. Ultimately, they exhibit almost constant values in the 1.5-3 MeV energy range, followed by a little increase to 15 MeV. The maximum Z_eff_ and N_el_ values of any sample that has been produced range from 11.398 to 2.9059 × 10^23^ (at 1.33 MeV) to 29.7902 and 7.5948 × 10^23^ (at 25 keV), respectively. Since higher Z_eff_ materials interact more with γ-rays, they are typically better for γ-ray shielding. As a result, the S7 sample appears to be a promising material for γ-ray shielding among the samples produced.

**Fig. 14:**
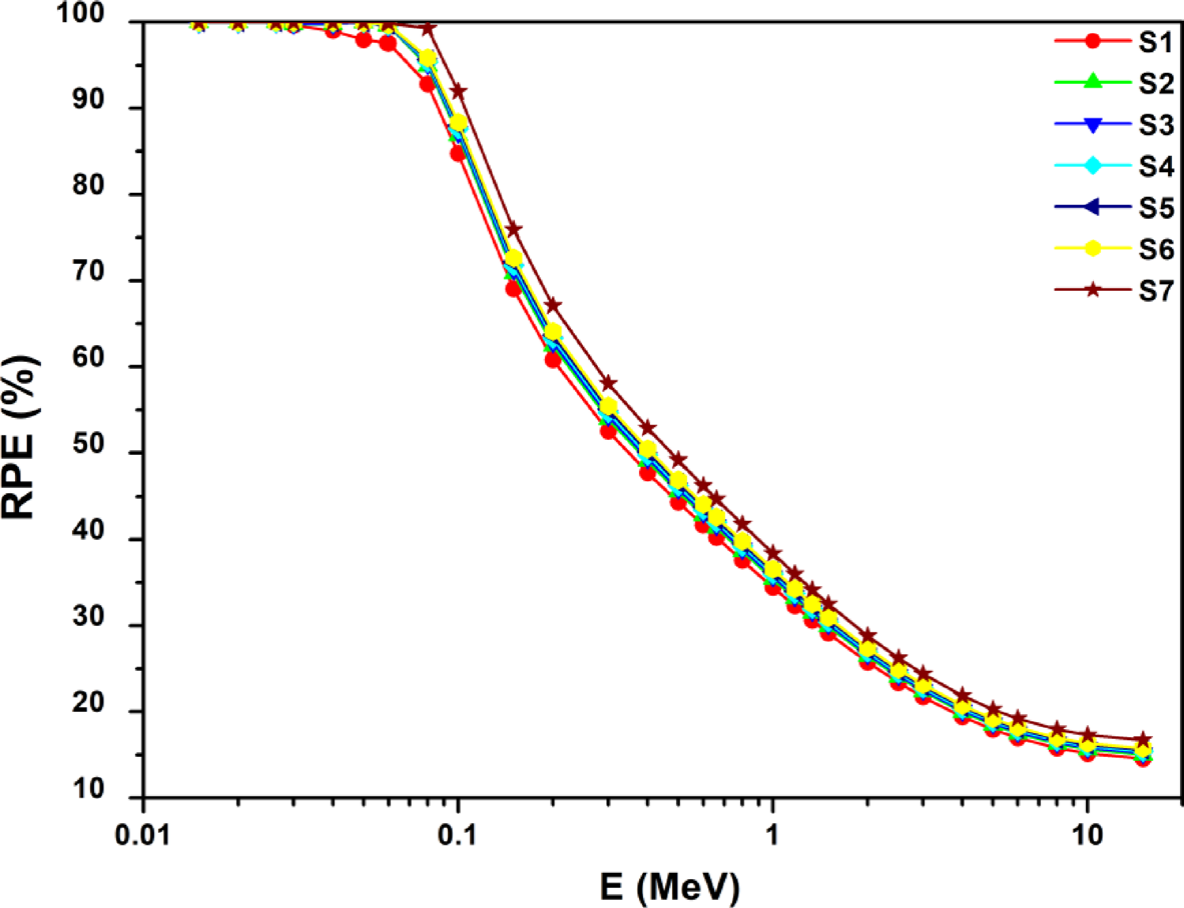
Variation of the predicted RPE values with photon energy for the produced glasses.

Moreover, for the manufactured glasses with 2 cm thick, the RPE (%) results were estimated and shown in Fig. 14 as a relative to photon energy. The results shown in Fig. 6 demonstrate that the S7 sample’s RPE provided 100% protection against X-ray photons with energy ranging from 30 to 100 keV. This means that the S7 sample needs to be employed in X-ray applications as a radiation protective material. The RPE of the S7 sample falls from its maximum value at 100 keV as the photon energy increases. It is also clear that gamma ray attenuation effectiveness is enhanced by increasing Mn concentrations.

**Fig. 15:**
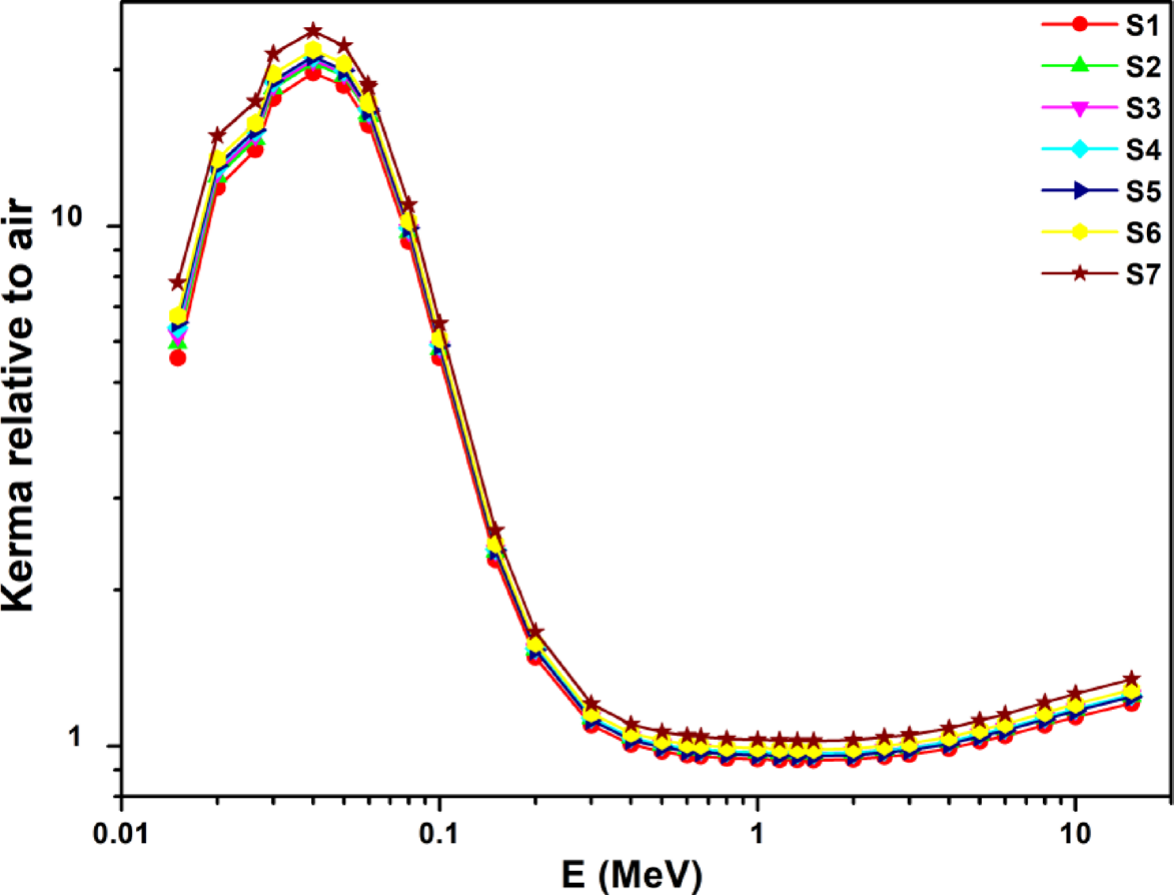
The current glasses’ Kerma changes in relation to air depending on the energy of gamma rays.

The kerma respect to air (Ka) dependency on energy for energy in the range of 15.0 keV to 15.0 MeV is shown in Fig. 15. The relative importance of the various photon-interaction processes is reflected in the connection between kerma and energy. A peak in the Ka values is produced by the PE process at the compound’s high-Z constituent’s K-absorption edge. The Ka values for Mn show a large peak about 0.04 MeV. Additionally, Fig. 15 demonstrates that the Ka not uniformly change with energy at energies below the K-absorption edge of the compound’s high-Z element, while there is a rapid reduction between 55 keV and 300 keV after the peak value. Between 300 keV and 3 MeV, Kerma is almost constant (Ka values of current glasses were independent of radiation energy), and then it steadily increases as energy increases.

###  Neutron attenuation characteristics

**Fig. 16:**
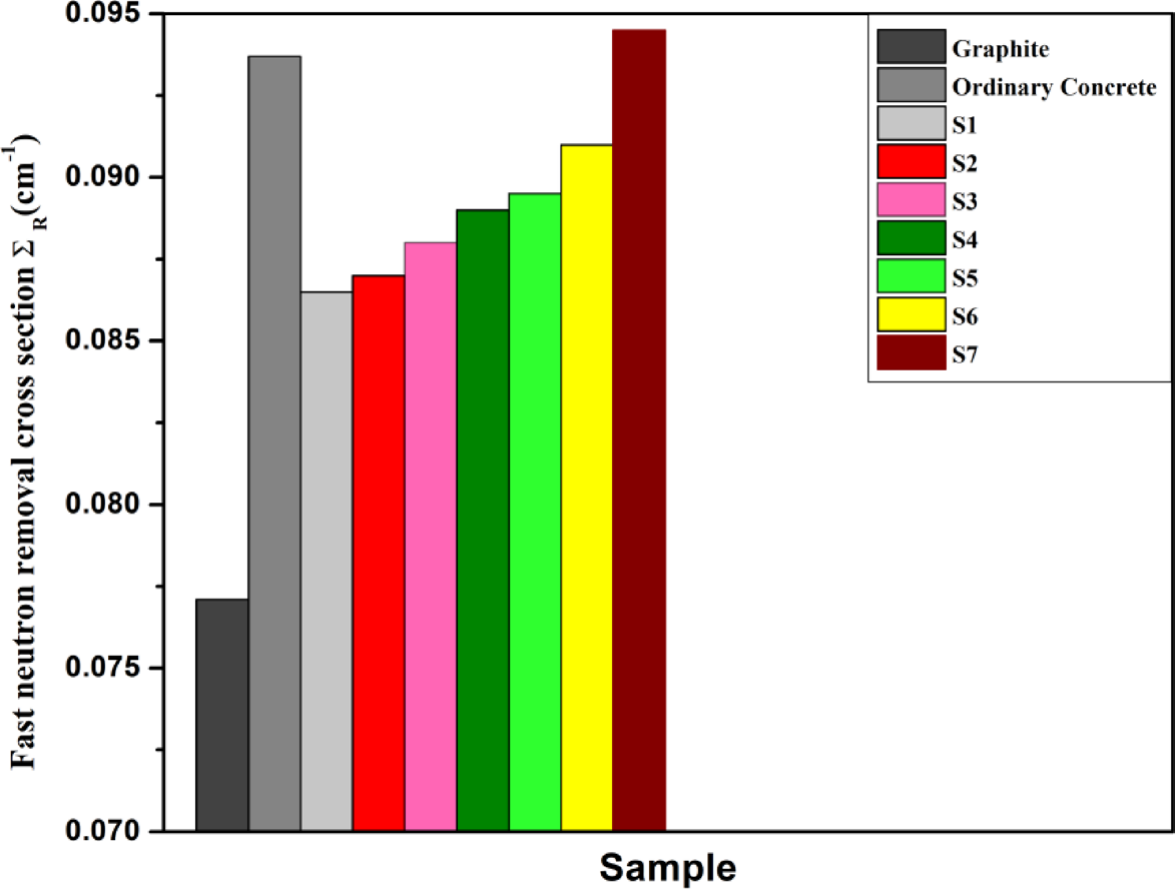
Effective removal cross section for fast neutrons of the present glasses along with that of other commonly used neutron shielding materials.

Equation 10 was used to assess the current glasses’ ∑_R_ values, which were found to range from 0.086 cm to 1 for S1 to 0.0956 cm-1 for S7. Furthermore, Fig. 16 compares the Σ_R_ of the current samples to that of typical neutron protection materials, like graphite and ordinary concrete.

The S7 sample has the uppermost value of Σ_R_ (0.0956 cm^− 1^) when associated to other frequently utilized neutron shielding materials, as Fig. 16 illustrates. This suggests that the S7 sample is a promising material candidate for fast neutron shielding applications.

### Protons and alpha particles’ shielding characteristics

**Fig. 17:**
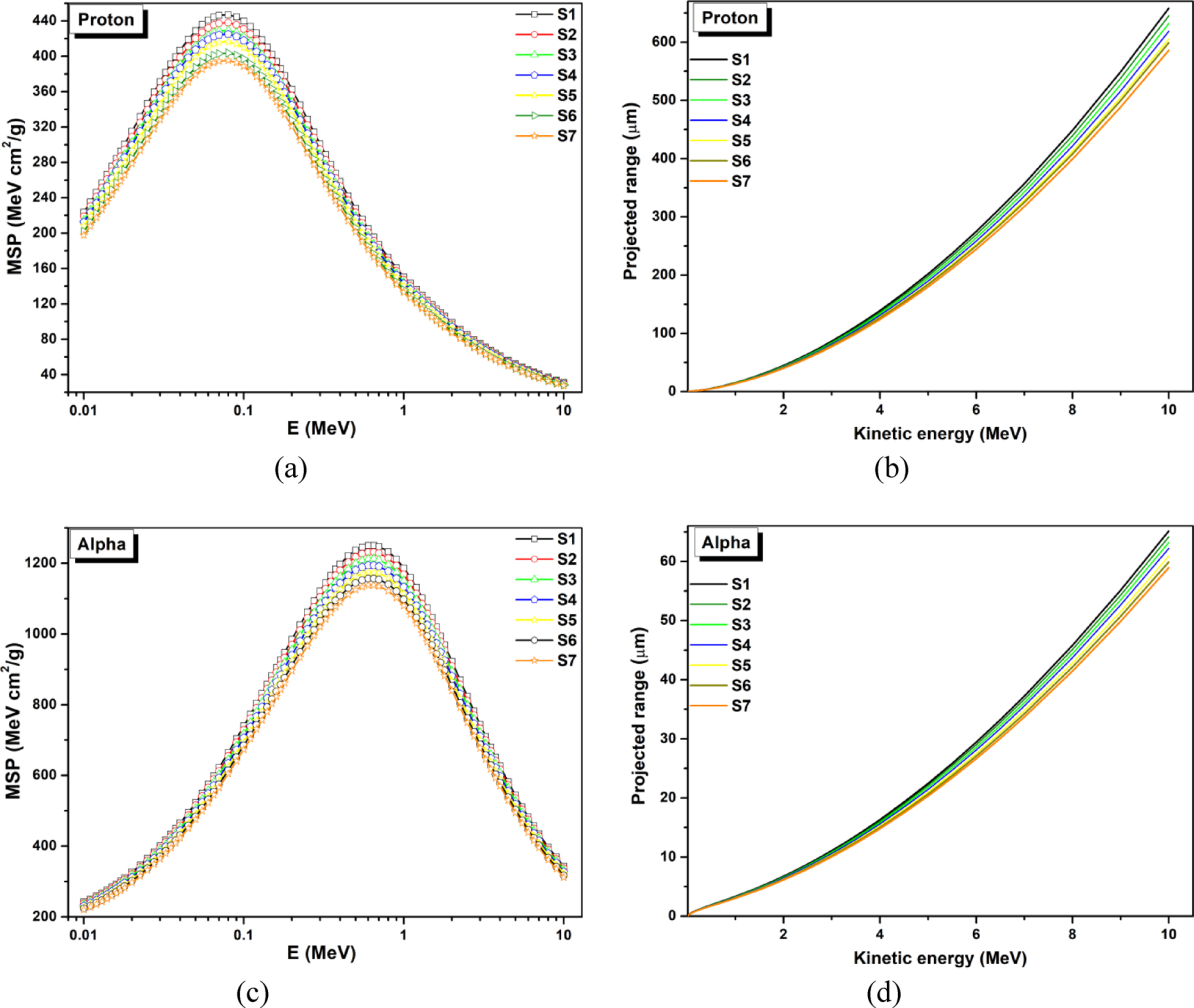
The anticipated ranges and MSP variations of the produced samples for protons and alpha particles (**a** &** b**) and (**c** &** d**) respectively in the energy range of 0.01–15 MeV.

Figure 17a, b) and 17c, d display the mass stopping power (MSP) and projected range (PR) of the generated glass’ protons (H^+ 1^) and alpha particles (He^+ 2^), respectively, as a function of ion kinetic energy between 0.01 and 15 MeV.

The statistics demonstrate that the projected ranges (PR) of the H^+ 1^ and He^+ 2^ ions rise as predicted by their kinetic energy. It is shown that the PR values for H^+ 1^ and He^+ 2^ ions improve for all samples in a nearly linear fashion as the particle kinetic energy increases. Additionally, the PR values for H^+ 1^ and He^+ 2^ ions are arranged into the following order for each energy line: Compared to S6, S5, S4, S3, S2, and S1, S7 is greater. More dense glass has lower PR values; this order is primarily based on the densities of the glass. It is crucial to remember that the PR values of the H^+ 1^ ion are roughly ten times greater than those of the He^+ 2^ ion for the same kinetic energy. The He^+ 2^ and H^+ 1^ ions’ disparate rest masses are the cause of this discrepancy. At the same energy, the H^+ 1^ ion has faster velocities than the He^+ 2^ ion because of its lower rest mass. Higher mass and velocity particles can penetrate more effectively because they have smaller interaction cross sections. The shortest PR values (which are desirable) for both H^+ 1^ and He^+ 2^ ions at the same energy are found in the S1 sample makes it special. The substantial contribution of Mn and the overall high density of the current glasses help to clarify this. Furthermore, for MSP values of protons and α-particles in the generated glasses. Higher atomic number glasses exhibit lower MSP values for H^+ 1^ and He^+ 2^ ions because atomic number and MSP are inversely correlated. Compared to other glasses under investigation, the S7 sample seems to be more efficient in absorbing protons (H + 1) and alpha particles (He^+ 2^) with the same kinetic energy.

## Conclusion

The goal of this work was to strike a fair compromise between the nuclear shielding and mechanical capabilities of seven glasses in the system xMnO-(50-x)P_2_O_5_-30Na_2_O-20Nb_2_O_5_, where x might be up to 7.5 mol%. Based on the findings of the calculations and observations, the following main conclusions might be drawn:

1. These XRD patterns demonstrate two broad humps without any sharp peaks, signifying that the amorphous nature of these glasses.
2. At lower concentrations, Mn^2+^ ions likely act as network modifiers, occupying interstitial sites. However, at higher concentrations (7.5%), the structural changes evident in the FTIR spectra suggest that manganese ions may partially enter the glass network, possibly in both Mn^2+^ and Mn^3+^ oxidation states, leading to the formation of MnO_4_ and MnO_6_ structural units. This dual role of manganese contributes to the observed changes in the glass network structure and the appearance of new vibrational modes at higher concentrations.
3. These show that the hardness and yield strength values were significantly improved by adding more MnO.
4. The creep strength was enhanced by adding more MnO.
5. The S7 sample is the most suitable sample for gamma-ray shielding among the glass samples being studied.
6. The S7 galss’s half-value layer (HVL) is substantially lower than the HVL values of all the other prepared samples and the HVL values discovered in previous studies of widely used radiation shielding materials, confirming the suitability of using the current glass as radiation shielding materials.
7. Gamma ray attenuation effectiveness is enhanced by increasing Mn concentrations.
8. The S7 sample has the highest value of Σ_R_ (0.0956 cm^− 1^) when compared to other widely utilized neutron shielding materials.
9. The S7 sample appears to be more effective in absorbing protons (H^+ 1^) and alpha particles (He^+ 2^) with the same kinetic energy when compared to other glasses that are being studied.

## Data Availability

Data will be made available from corresponding author on request.
